# Legumain Activity Is Controlled by Extended Active Site Residues and Substrate Conformation

**DOI:** 10.3390/ijms232012548

**Published:** 2022-10-19

**Authors:** Tasneem Elamin, Hans Brandstetter, Elfriede Dall

**Affiliations:** Department of Biosciences and Medical Biology, University of Salzburg, 5020 Salzburg, Austria

**Keywords:** asparaginyl endopeptidase, protein structure, pH-dependent activity

## Abstract

Legumain is a lysosomal cysteine protease with strict specificity for cleaving after asparagine residues. By sequence comparison, legumain belongs to MEROPS clan CD of the cysteine proteases, which indicates its structural and mechanistic relation to caspases. Contrasting caspases, legumain harbors a pH-dependent ligase activity in addition to the protease activity. Although we already have a significant body of knowledge on the catalytic activities of legumain, many mechanistic details are still elusive. In this study, we provide evidence that extended active site residues and substrate conformation are steering legumain activities. Biochemical experiments and bioinformatics analysis showed that the catalytic Cys189 and His148 residues are regulated by sterically close Glu190, Ser215 and Asn42 residues. While Glu190 serves as an activity brake, Ser215 and Asn42 have a favorable effect on legumain protease activity. Mutagenesis studies using caspase-9 as model enzyme additionally showed that a similar Glu190 activity brake is also implemented in the caspases. Furthermore, we show that the substrate’s conformational flexibility determines whether it will be hydrolyzed or ligated by legumain. The functional understanding of the extended active site residues and of substrate prerequisites will allow us to engineer proteases with increased enzymatic activity and better ligase substrates, with relevance for biotechnological applications.

## 1. Introduction

Proteases are important signaling molecules that are involved in virtually all biological processes in all living organisms. By consequence, dysregulation of their protease activity can have severe effects resulting in a variety of pathologies, including cancer and Alzheimer’s disease [1,2]. Therefore, proteases must be regulated delicately and on different levels. The cysteine protease legumain is an important player of our immune system. It is primarily localized to the endo-lysosomal system, where its main function is the processing of (foreign)antigens for subsequent presentation on the MHCII complex [3,4]. Moreover, it proteolytically activates Toll-like receptors, and endo-lysosomal cysteine cathepsins [5,6]. Due to its strict specificity for cleaving after asparagine residues, it is synonymously termed the asparaginyl endopeptidase (AEP) [7,8]. In addition to its well-known protease activity, legumain also harbors a ligase activity [9,10]. Protease and ligase activity are in a pH-dependent equilibrium with the protease dominating at acidic pH and the ligase at neutral pH. Physiologic substrates of its ligase function include legumain itself and its endogenous inhibitor human cystatin M/E (hCE) [9,11]. Importantly, the protease and ligase activity of legumain use the same active site, which comprises the catalytic triad Cys189-His148-Asn42 [11]. On a pathophysiological level, legumain is overexpressed in the majority of human solid tumors, including breast cancer, colorectal cancer and gastric cancer [12,13,14,15]. Under these conditions, legumain is associated with enhanced tissue invasion and metastasis and consistently overexpression correlates with poor prognosis. Moreover, legumain was also linked to the aggregation of proteins critically linked with neurodegenerative diseases, finally causing neuronal damage [16,17]. Not only, but especially because of its pathophysiologic function, legumain activity must be regulated tightly and on different levels. Its activity is controlled by its synthesis as a zymogen form which needs to undergo auto-catalytic activation at acidic pH, by endogenous inhibitors of the cystatin family, by conformational destabilization of mature legumain at neutral pH, and by its pH-dependent protease and ligase activities [18,19]. Although we already understand the fundamentals of legumain’s catalytic activity, many mechanistic details are still elusive. A hallmark of legumain-like clan CD proteases is the steric separation of the catalytic cysteine and histidine residues by the substrate [20]. Consequently, the catalytic residues cannot directly interact with each other, as found in serine proteases or the papain-like clan CA cysteine proteases [21]. We hypothesize that instead the catalytic residues are regulated by extended active site residues. In a previous study we found that legumain’s activity is indeed modulated by residue Glu190, which is located directly next to the catalytic Cys189 residue [22]. Glu190 decreases the enzymatic activity of legumain by stabilizing the protonated state of the Cys189 Sγ atom. Thereby it reduces the nucleophilicity of Cys189 and hence substrate hydrolysis. Additionally, we found that the ligase function of legumain is critically dependent on the affinity of the prime-side substrate [10]. Together, these observations led to the hypothesis that the enzymatic activity of legumain is not only controlled by the catalytic residues but also by other regulatory factors that are located on the enzyme and the substrate. Therefore, we set out to study enzyme- and substrate-dependent aspects of legumain’s activity regulation. Specifically, we analyzed the function of extended active site residues and of substrate conformation for legumain activity. These studies led to a better understanding of the catalytic activity of legumain and of clan CD proteases in general.

## 2. Results

### 2.1. Asn42 Is Essential for Legumain Protease Activity but Less for Its Ligase Activity

**Figure 1:**
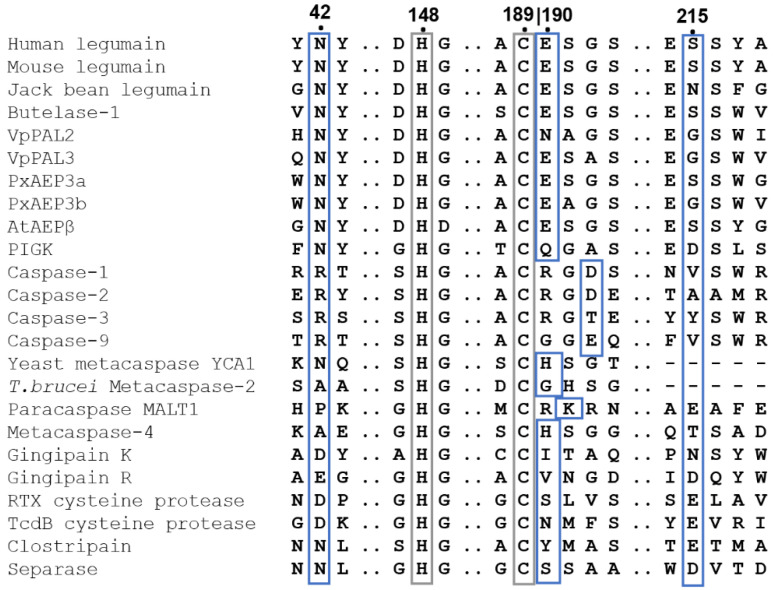
Structure-based sequence alignment of clan CD proteases revealed strict conservation of catalytic cysteine and histidine residues and protease specific regulatory residues. The catalytic Cys189 and His148 (human legumain numbering) are indicated by a grey box. Regulatory residues are indicated by blue boxes. The alignment was prepared based on structures of the following clan CD proteases: human legumain (pdb 7O50), mouse legumain (pdb 4NOJ), jack bean legumain (pdb 6XT5), butelase-1 (pdb 6DHI), PIGK (phosphatidylinositol glycan anchor biosynthesis class K protein, pdb 7W72), caspase-1 (pdb 1ICE), caspase-2 (pdb 1PYO), caspase-3 (pdb 1CP3), caspase-9 (pdb 1NW9), yeast metacaspase-1 (YCA1, pdb 4F6O), *T. brucei* metacaspase-2 (pdb 4AFP), *A. thaliana* metacaspase-4 (pdb 6W8R), human paracaspase MALT1 (pdb 3UOA), *P. gingivalis* gingipain K (pdb 4RBM), *P. gingivalis* gingipain R (pdb 1CVR), *V. cholerae* RTX cysteine protease (pdb 3EEB), *C. difficile* TcdB cysteine protease (pdb 3PEE), *P. merdae* clostripain (pdb 4YEC) and human separase (pdb 7NJ1). Furthermore, sequences of the peptide asparaginyl ligase 2 and 3 from *Viola philippica* (VpPAL2 and VpPAL3) were included.

**Figure 2:**
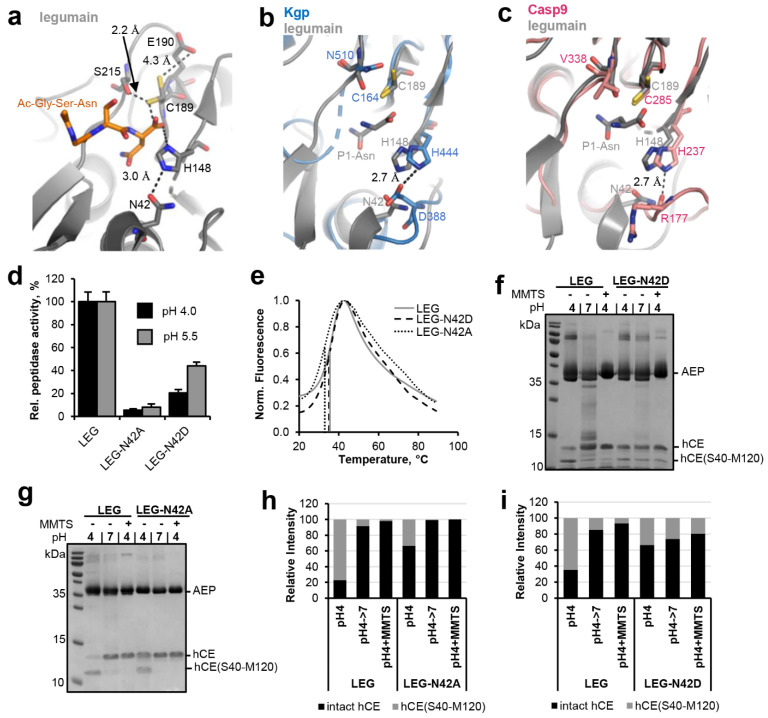
Asn42 enhances legumain protease activity. (**a**) Zoom-in view on the active site of legumain in complex with the Ac-Gly-Ser-Asn peptide (GSN peptide; pdb 7o50). Active site residues are shown in grey sticks, the peptide ligand in orange sticks. Please note that the side chain of Cys189 adopted two alternative conformations within this structure. (**b**) Superposition of the active sites of legumain (grey, pdb 7o50) and *P. gingivalis* gingipain K (blue, pdb 4rbm). (**c**) Superposition of the active site of legumain (grey) and caspase-9 (pink, pdb 1jxq). Active site residues are shown in sticks. The P1-Asn residue (grey sticks) of the GSN peptide indicates the position of the S1 specificity pocket. (**d**) Activity of wild-type, N42A- and N42D-legumain (LEG) measured as turnover of the Bz-Asn-pNA substrate at pH 4.0 and pH 5.5. (**e**) Thermal unfolding of wild-type (LEG), N42A- and N42D-legumain (LEG-N42A and LEG-N42D), measured at pH 6.0. (**f**,**g**) Incubation of legumain with cystatin M/E (hCE; molar ratio 3:1) at pH 4 led to processing after the P1-Asn39 residue. The C-terminal cleavage product hCE(S40-M120) is indicated. Subsequent incubation at pH 7.0 or addition of S-methyl methanethiosulfonate (MMTS) led to resynthesis of the intact cystatin M/E by re-ligation of the Asn39-Ser40 peptide bond. MMTS leads to a covalent thiomethylation of the catalytic Cys189. (**h**,**i**) Bands corresponding to intact cystatin M/E and to the C-terminal cleavage product hCE(S40-M120) shown in (**f**,**g**) were quantified using ImageJ.

Legumain’s enzymatic activity is critically dependent on its active site, which is formed by the catalytic triad Cys189-His148-Asn42. While structure-based sequence alignments showed that Cys189 and His148 are strictly conserved within the clan CD proteases, we found that Asn42 is variable (Figure 1). *P. gingivalis* gingipain K harbors an aspartate residue at the structurally equivalent position (Figure 2a,b). Caspases, paracaspase MALT1 and metacaspases (metacaspase 2, 4) harbor a carbonyl oxygen (e.g., Arg177 in caspase-9) in place of Asn42 (Figure 1 and Figure 2c). This variation in the active site residues is remarkable and made us hypothesize that residue 42 has a protease-specific regulatory function. To test this hypothesis, N42A- and N42D-legumain variants were prepared and their activity towards the legumain-specific Bz-Asn-pNA substrate was measured at pH 4.0 and 5.5. Importantly, we found that the N42A-legumain mutant had a dramatically reduced residual activity of <10% at pH 4.0 and 5.5 as compared to the wild-type enzyme (Figure 2d). This result clearly shows that the side chain of residue 42 is essential for the protease activity of legumain. A similar mutation in mouse legumain also abolished legumain protease activity [9]. Interestingly, the N42D-legumain variant showed a pH-dependent reduction in activity. While it preserved about 50% residual activity at pH 5.5, its residual activity was reduced to approx. 20% at pH 4.0. This finding suggests that if residue 42 was an aspartate, as found in gingipain K, it needs to be deprotonated for efficient catalysis. This conclusion is also consistent with the pH-activity optimum of gingipain K at around neutral pH [23]. To rule out that the reduction in activity was due to conformational destabilization by the introduced mutations, the thermal stability of the N42A- and N42D-legumain mutants was tested and compared to wild-type legumain. Indeed, we found that the thermal stability of the N42A and N42D mutants was similar to wild-type legumain (Figure 2e). Thereby it can be excluded that the reduction in protease activity was due to conformational destabilization.

In a next step we set out to test the relevance of Asn42 for the ligase activity of legumain. To that end, human cystatin M/E (hCE) was used as a model substrate. In a previous study we found that cystatin M/E is processed by legumain after the P1-Asn39 residue on its reactive center loop (RCL), which results in an N-terminal hCE(M1-N39) and a C-terminal hCE(S40-M120) cleavage product [11]. Importantly, the N- and C-terminal fragments do not dissociate upon cleavage. Only under denaturing conditions, as used for example for SDS-PAGE experiments, cleaved cystatin M/E will unfold and the cleavage products will separate. Processing of cystatin M/E occurred at pH ≤ 5.0 and was reversible upon shifting pH to near neutral or upon covalently blocking the catalytic Cys189 by the addition of MMTS (S-methyl methanethiosulfonate). MMTS led to a covalent reversible thiomethylation of the catalytic Cys189, which prevents the formation of the covalent thioester intermediate. Therefore, modification with MMTS will block legumain’s protease activity (Figure S1). However, we previously found that cysteine modification has a beneficial effect on the ligase activity of legumain, where free termini are joined without the requirement of a covalent thioester intermediate [11]. When the catalytic Cys189 is blocked, the Asn39-Ser40 peptide bond of pre-cleaved cystatin M/E is rejoined by legumain and will remain stable because Cys189 will not be able to form a covalent thioester intermediate which is required for subsequent hydrolysis. To test the relevance of Asn42 for legumain’s ligase activity the N42A- and N42D-legumain mutants were incubated with cystatin M/E at pH 4.0 and its processing was monitored on SDS-PAGE (Figure 2f,g). Subsequently, pH was shifted to 7.0 or MMTS was added to trigger re-ligation of the inhibitor. In control experiments wild-type legumain was used instead. In agreement with our Bz-Asn-pNA turnover experiments, we found less processing of cystatin M/E by the N42A-legumain mutant as compared to wild-type legumain (Figure 2g,h). However, the cleavage product was efficiently ligated by legumain upon shifting pH to 7.0 or by adding MMTS. The N42D-legumain mutant similarly showed a reduction in cystatin M/E hydrolysis (Figure 2f,i). Contrasting N42A-legumain, N42D-legumain showed a significant reduction in its ligase activity. Together these experiments showed that Asn42 is a critical residue for the protease and ligase activity of legumain. Asn42 is in hydrogen bonding distance to the catalytic His148 residue and might indirectly facilitate the protonation of the substrate’s amine by orienting the catalytic histidine residue (Figure 2a).

### 2.2. Fine-Tuning of Clan CD Protease Activity Is Achieved by pKa Modulation of the Catalytic Cysteine Residue

**Figure 3:**
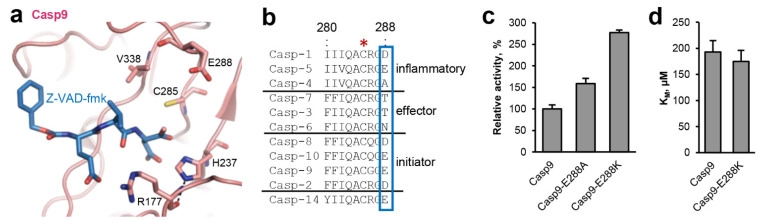
A pKa tuning switch is also implemented in caspases. (**a**) Zoom-in view on the active site of caspase-9 (pdb 1jxq). Active site residues are shown in pink sticks, the Z-VAD-fmk inhibitor is shown in blue sticks. (**b**) Sequence alignment of human caspases. The catalytic Cys285 residue is indicated by a red star. The pKa switch at position 288 is highlighted by a blue box. Uniprot IDs are specified in the material and methods section. (**c**) Activity of ΔCARD-caspase-9 and the ΔCARD-caspase-9-E288K mutant measured as turnover of the VAD-AMC substrate. (**d**) K_M_ values of ΔCARD-caspase-9 and the ΔCARD-caspase-9-E288K mutant determined using the VAD-AMC substrate.

Within the active site of legumain the catalytic Cys189 and His148 residues are separated by the substrate, preventing them from direct interaction with each other (Figure 2a). Instead, our studies suggest that the catalytic residues are regulated by an extended network of sterically inter-connected, active site residues. We showed that His148 is modulated by Asn42 during catalysis. Analogous, we found residues Glu190 and Ser215 in close proximity to the catalytic Cys189 residue (Figure 2a). This colocalization led to the hypothesis that Glu190 and Ser215 will have a regulatory effect on Cys189, similar as we saw it in Asn42-His148. Indeed, we could previously show that Glu190 has a pKa tuning effect on Cys189. It stabilizes the protonated state of Cys189, thereby reducing its nucleophilicity and consequently substrate hydrolysis rate [22]. Prompted by this observation, we wondered whether the pKa tuning mechanism was conserved within the clan CD proteases. Analyzing structures and sequences of the related caspases, we found that they harbor residue 288 (caspase-9 numbering) at a structurally similar position (Figure 1 and Figure 3a,b). However, unlike Glu190, which is highly conserved in legumain, residue 288 is variable in caspases. In most caspase isoforms we found a negatively charged aspartate or glutamate at the equivalent position (Figure 3b), which indicated that a similar activity brake was also present in caspases. However, we also observed alanine, threonine or asparagine residues in caspase-4, 7, 3, and 6. To test whether the pKa tuning mechanism was indeed also implemented in the caspases, caspase-9 was used as a model enzyme. Specifically, ΔCARD caspase-9-E288K charge reversal and ΔCARD caspase-9-E288A charge neutralization mutants were prepared. According to our hypothesis both mutants should release the activity brake implemented by Glu288. Indeed, when the enzymatic activity of the ΔCARD caspase-9-E288A mutant towards the VAD-AMC substrate was tested, we found an increase in activity by approx. 50% (Figure 3c). Importantly, the ΔCARD caspase-9-E288K charge reversal mutant showed an even higher increase in activity of about 200% compared to the wild-type enzyme. To test whether this effect was indeed related to catalytic efficiency (kcat) rather than substrate affinity, K_M_ values of wild-type ΔCARD caspase-9 and the ΔCARD caspase-9-E288K mutant towards the VAD-AMC substrate were determined. Indeed, both caspase-9 variants showed a similar K_M_ value (Figure 3d). This result indirectly confirms that the effect we observed was not caused by a change in substrate affinity but by a difference in catalytic efficiency (kcat). Since we knew that the Glu190 pKa tuning mechanism was implemented not only in legumain but in general in the clan CD proteases, we hypothesized that some members may take advantage of it, by presenting a positively charged amino acid at the respective position. Indeed, when we analyzed the crystal structures and sequences of paracaspase Malt1 (pdb 4i1p) and of metacaspase-4 (*Arabidopsis thaliana*, pdb 6w8t) we found that they harbor Lys466 and His140, respectively, at a position equivalent to Glu190 in legumain (Figure 1). Based on this observation, we suggest that they use pKa tuning to improve the nucleophilicity of the catalytic cysteine residue.

### 2.3. Ser215 Is Extending the Legumain Active Site

**Figure 4:**
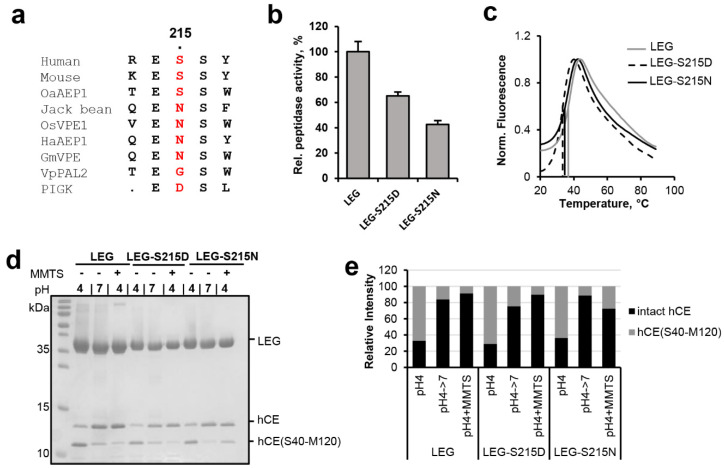
Ser215 is enhancing legumain protease activity towards peptidic substrates. (**a**) Sequence alignment of human, mouse, jack bean, *O. sativa* (rice), and *H. annuus* (sunflower) legumain and the PIGK protein (phosphatidylinositol glycan anchor biosynthesis class K protein, part of the glycosylphosphatidylinositol transamidase complex). Residue 215 (human legumain numbering) is highlighted in red. Uniprot IDs of the sequences used are specified in the material and methods section. (**b**) Relative activity of wild-type legumain (LEG), and the legumain-S215N and -S215D mutants measured as turnover of the AAN-AMC substrate at pH 5.5. (**c**) Thermal unfolding of wild-type (LEG), S215A- and S215D-legumain variants (LEG-S215A and LEG-S215D), measured at pH 6.0. (**d**) Incubation of legumain with cystatin M/E (hCE) at pH 4.0 led to processing of cystatin M/E after the P1-Asn39 residue. The C-terminal cleavage product hCE(S40-M120) is indicated. Subsequent incubation at pH 7.0 or addition of S-methyl methanethiosulfonate (MMTS) led to resynthesis of the intact cystatin M/E by re-ligation of the Asn39-Ser40 peptide bond. MMTS leads to a covalent thiomethylation of the catalytic Cys189. (**e**) Bands corresponding to intact cystatin M/E and to the C-terminal cleavage product hCE(S40-M120) shown in (**d**) were quantified using ImageJ.

Since Ser215 was, like Glu190, close to the catalytic Cys189 residue, we next wondered whether it would also have a regulatory effect on legumain activity. Sequence analysis showed that Ser215 was highly conserved within the family C13 legumain-like proteases. However, we found specific variations in legumain sequences with proven ligase activity, including jack bean (*Canavalia ensiformis*), rice (*Oryza sativa*), and sunflower (*Helianthus annuus*) legumain and the PIGK protein (phosphatidylinositol glycan anchor biosynthesis class K protein, part of the glycosylphosphatidylinositol transamidase complex), which harbor an asparagine or aspartate residue at the respective position (Figure 1 and Figure 4A). To find out how these variations would affect legumain protease activity, S215D- and S215N-legumain mutants were prepared and their activity towards the AAN-AMC substrate was tested. Interestingly, we found that the S215D-legumain mutant showed only 60% residual activity towards the AAN-AMC peptide substrate (Figure 4b). This effect indicated that in the S215D-legumain mutant a pKa tuning mechanism contributed to the activity, similar as in Glu190. Importantly, thermal shift experiments confirmed that the thermal stability of the S215D-legumain mutant was similar to wild-type legumain (Figure 4c). This experiment clearly showed that the reduction in enzymatic activity was not related to conformational destabilization caused by the point mutation. The S215N-legumain variant showed an even more pronounced reduction in activity, with only approx. 40% residual activity left (Figure 4b). This reduction in activity was comparable to a S215A-legumain variant which we also recently analyzed [10]. Similar to the S215D-legumain variant, the S215N variant also showed a wild-type-like thermal stability, which confirmed its conformational integrity. Since not only the S215D-legumain mutant, but also the S215N-legumain mutant showed a reduction in protease activity, we concluded that the effect of Ser215 might not primarily be on the protonation of Cys189 but on its orientation. When different structures of human legumain were compared, we found that the side chains of the catalytic Cys189 and of Ser215 were flipping between two distinct orientations (Figure 2a and Figure S2). Since Cys189 and Ser215 are in hydrogen bonding distance, we assume that they undergo a concerted side chain reorientation. Ser215 may assist in positioning the Cys189 Sγ during catalysis. Importantly, our study shows that legumain variants carrying asparagine (jack bean legumain) or aspartate (PIGK) residues at position 215 harbor a reduced protease activity. Interestingly, Ser215 is replaced by valine, tyrosine, alanine or isoleucine residues in the caspases (e.g., Val338 in caspase-9), by asparagine 510 in gingipain K and by aspartate or glutamate residues in Malt-1, gingipain R, the RTX cysteine protease, the TcdB cysteine protease, clostripain and separase (Figure 1). This sequence variation suggests a similar reduction in activity within these proteases.

In a next step the effect of the S215N/D variations on legumain ligase function was analyzed. Again, cystatin M/E was used as a model substrate. Both mutants showed a wild-type-like hydrolysis of cystatin M/E at pH 4.0 and were able to re-ligate it (Figure 4d,e).

### 2.4. Legumain’s Enzymatic Activity Is Controlled by Substrate Conformation

**Figure 5:**
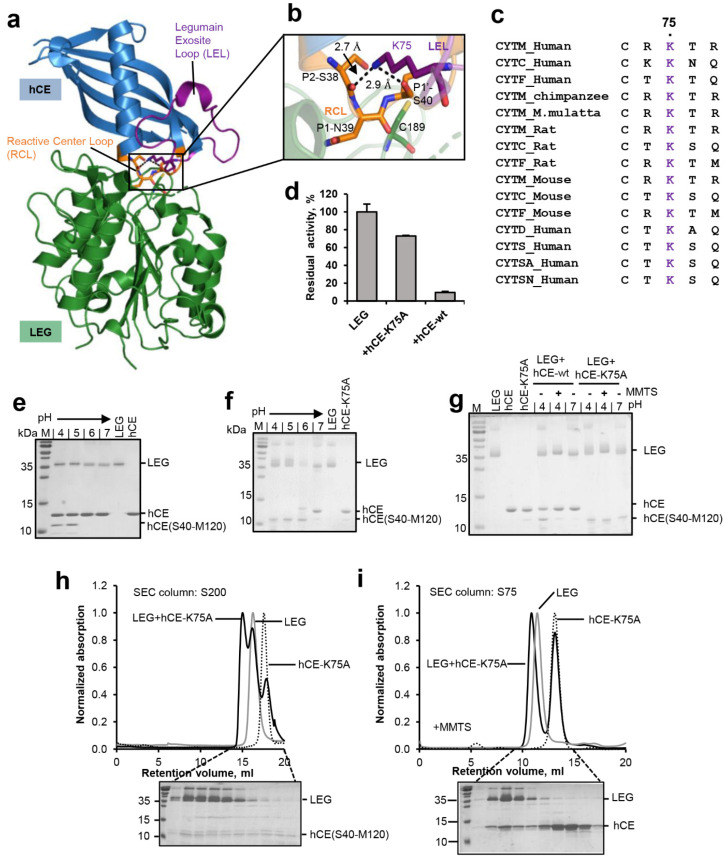
Substrate conformation is critical for hydrolysis and ligation. (**a**) Structure of legumain (LEG, green) in complex with cystatin M/E (hCE, blue, pdb 4n6o). The reactive center loop (RCL) is shown in orange, the legumain exosite loop (LEL) in purple. (**b**) Zoom-in view on the RCL (orange). The catalytic Cys189 is indicated in green sticks, the P2–P1′ residues on the RCL as orange sticks and Lys75 on the LEL as purple sticks. (**c**) Sequence alignment of family 2 cystatins C, D, M/E, F, S, SA, and SN. Lys75 is highlighted in purple. (**d**) Residual activity of legumain measured as turnover of the AAN-AMC substrate upon incubation with wild-type cystatin M/E (hCE-wt) or the cystatin M/E-K75A (hCE-K75A) mutant at pH 5.5. (**e**) SDS-PAGE gel after incubating legumain with wild-type cystatin M/E at indicated pH values for 1 h at 37 °C. (**f**) Same as (**e**) but using the cystatin M/E-K75A mutant. The C-terminal cleavage product hCE(S40-M120) is indicated. (**g**) Incubation of legumain with a 4-fold molar excess of cystatin M/E or the cystatin M/E-K75A mutant at pH 4.0 led to processing of the cystatin after the P1-Asn39 residue. Subsequent incubation at pH 7.0 or addition of S-methyl methanethiosulfonate (MMTS) led to re-ligation of the Asn39-Ser40 peptide bond in wild-type cystatin M/E but not in the cystatin M/E-K75A mutant. (**h**) Legumain was co-incubated with the cystatin M/E-K75A mutant at pH 4.0 in a 1:4 molar ratio and subjected to size exclusion chromatography (SEC) at pH 4.0. In control experiments legumain alone (LEG) or cystatin M/E-K75A alone were loaded onto the SEC column. (**i**) Same as (**h**) but legumain was pre-incubated with MMTS to covalently block the catalytic Cys189 and thereby prevent cleavage of the cystatin. Indicated peak fractions of the experiment containing both legumain and cystatin M/E-K75A were analyzed by SDS-PAGE.

Our previous studies indicated that it is not only the active site of legumain that is critical for its activity, but that efficient proteolysis and ligation also depend on features of the substrate. Specifically, we found that substrate affinity governs the equilibrium between proteolysis and ligation [10]. Experiments using peptidic substrates showed that a substrate’s affinity depends on its amino acid sequence. We expect however that a substrate’s conformation will be as important for efficient proteolysis and ligation by legumain. To test this hypothesis, cystatin M/E was used as a model substrate. Cystatin M/E harbors a rigid RCL conformation, which is established by a conserved Lys75 located on the legumain exosite loop (LEL, Figure 5a–c). Specifically, Lys75 mediates hydrogen bonds to the carbonyl oxygen of P2-Ser38 and the Oγ of P1′-Ser40. Mutation of Lys75 to alanine will abolish these stabilizing interactions, which allows us to specifically control the RCL conformation. Along that line, a cystatin M/E-K75A (hCE-K75A) mutant was prepared and its interaction with legumain was tested. Interestingly, we found that the cystatin M/E-K75A mutant showed a reduction in legumain inhibition, as compared to the wild-type inhibitor (Figure 5d). This reduction in inhibitor potency suggested that the rigid RCL conformation is critical for inhibition. To test the susceptibility of the RCL for legumain protease activity, wild-type cystatin M/E and the cystatin M/E-K75A mutant were incubated with legumain at pH 4.0 to 7.0 in a 2:1 molar ratio. While wild-type cystatin M/E was only processed to <50% at pH 4.0–5.0, the K75A mutant was completely processed by legumain at pH 4.0–6.0 (Figure 5e,f). The increased turnover of cystatin M/E-K75A showed that the increase in RCL flexibility favored its hydrolysis and release from the active site, thereby demonstrating that the flexibility of the substrate is an important prerequisite for efficient processing. To test the effect of RCL flexibility on legumain’s ligase activity, wild-type cystatin M/E or the cystatin M/E-K75A mutant were co-incubated with legumain at pH 4.0 until >50% RCL processing was achieved, as judged by SDS-PAGE. Subsequently pH was shifted to 7.0 or MMTS was added to trigger the ligase reaction. Importantly, we found that legumain was not able to re-ligate the cystatin-K75A mutant (Figure 5g). This observation suggested that Lys75 is critical to maintain the prime and non-prime cleavage products in close proximity, to allow for subsequent re-ligation. Importantly, Lys75 is strictly conserved within the family 2 cystatins (Figure 5c), which further corroborates its mechanistic importance for legumain inhibition.

In a next step, we wanted to test whether processed cystatin M/E-K75A still binds to legumain after cleavage. To that end, the cystatin M/E-K75A mutant was co-incubated with legumain at pH 4.0 till complete RCL hydrolysis was achieved, as judged by SDS-PAGE. Subsequently, this sample was loaded on a size exclusion column pre-equilibrated in buffer at pH 4.0. Indeed, we found a peak migrating at higher molecular weight as compared to legumain alone (Figure 5h). As expected, SDS-PAGE analysis of the peak fractions showed that the cleaved cystatin M/E-K75A inhibitor was co-migrating with legumain. However, the molar ratio of enzyme:inhibitor within this peak was >1:1. This observation led to the conclusion that (1) the hCE-K75A mutant was at least partially degraded by legumain during the size exclusion run and that (2) cleavage of the cystatin M/E-K75A mutant had a negative effect on its affinity to legumain. To further analyze these hypothesis, legumain was pre-incubated with MMTS before the cystatin M/E-K75A mutant was added. MMTS will covalently block the catalytic cysteine residue and thereby prevent it from cleaving the inhibitor. Due to its small size, MMTS is sterically compatible with cystatin binding [11]. Indeed, when the catalytic Cys189 residue was blocked, we observed an increase in co-migration of intact cystatin M/E-K75A inhibitor with legumain (Figure 5i). This result is in line with our hypothesis that processed cystatin M/E-K75A had a lower affinity to legumain as compared to the intact cystatin. However, given that significant amounts of intact cystatin M/E-K75A also migrated separate from legumain we concluded that (i) the reactive center loop-destabilized cystatin M/E-K75A variant had a reduced affinity towards legumain as compared to wild-type cystatin M/E, and (ii) the cleavage of cystatin M/E-K75A aggravated this effect. Along the same line, an affinity constant for the inhibition of legumain by the cystatin M/E-K75A mutant was determined (Figure S3). Consistent with our size exclusion experiments, we measured a *K_i_* value of 19.8 nM ± 2.8 nM, which is approx. 1000 times higher as compared to the *K_i_* of wild-type cystatin M/E (1.6 pM) [19].

## 3. Discussion

**Figure 6:**
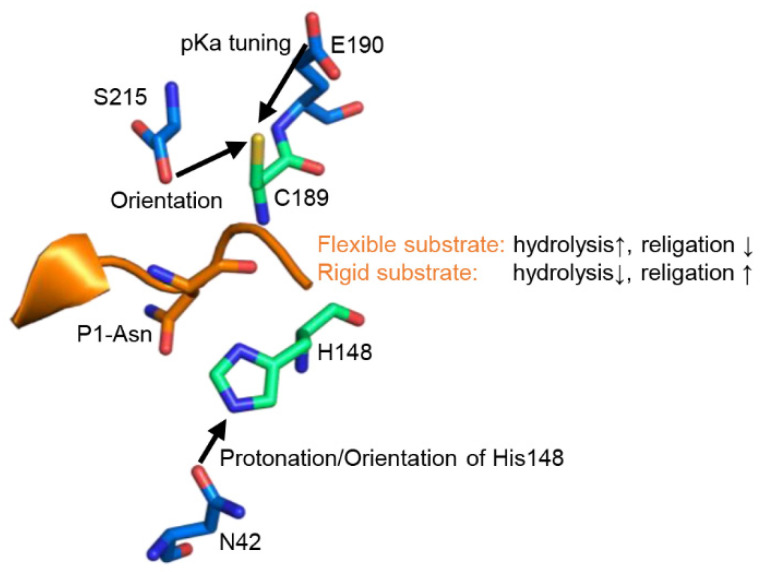
Legumain activities are regulated by extended active site residues and substrate conformation. Zoom-in view on the active site of legumain as seen in the crystal structure of legumain in complex with cystatin M/E (pdb 4n6o). The catalytic Cys189 and His148 residues are shown in green sticks, regulatory residues on legumain in blue sticks. The position of the substrate is indicated by the reactive center loop of cystatin M/E (orange).

A remarkable feature of clan CD proteases is the separation of the catalytic cysteine and histidine residues by the substrate, which prevents their direct regulatory interaction. In the present study, we show that instead, the catalytic residues are regulated by extended active site residues and by substrate conformation. Specifically, we show that His148 is regulated by Asn42 and Cys189 by Glu190 and Ser215 (Figure 6). Importantly, both legumain and most caspases implemented a negatively charged residue in close proximity to the catalytic cysteine residue (position 190), whereby their protease activities are reduced. In the first place, introducing such a pKa-based activity brake seems surprising. However, it nicely shows that proteases are not optimized for maximum activity but for a specific level of activity. A good enzyme implements the right balance of activation and “inhibition”. While legumain installed an activity brake at position 190, the paracaspase Malt 1 and metacaspase-4 harbor a positively charged accelerator at the equivalent position. Thereby residue 190 allows enzyme specific tuning of protease activity. Although Glu190 is highly conserved in legumain sequences from different kingdoms of life, we found an E190N variation in *Violaceae* (VpPAL2, Figure 1), which suggests higher legumain protease activity within this plant family. However, the stimulating E190N variation is accompanied by an additional S215G variation, which we expect to counterbalance the Asn190 effect. Similar to Glu190, we found that variations at position 215 are also tuning legumain activity. Specifically, asparagine or aspartate residues at position 215, as we find it in jack bean legumain or the PIGK protein, led to an approx. 50% reduction in protease activity when introduced in legumain, which usually harbors a serine at this position. The mutations did not, however, cause a reduction in legumain ligase activity. The crystal structure of legumain in complex with an Ac-Gly-Ser-Asn peptide (pdb 7o50, Figure 2a) revealed that the Sγ atom of Cys189 exists in two distinct orientations, which favor protease or ligase reactions, respectively [10]. Likewise, superposing different legumain structures showed that the side chain of Ser215 also switches between two distinct conformations (Figure S1). Based on our present mutagenesis data, we suggest that the S215D variation may have a pKa tuning effect similar to Glu190. In addition, residue 215 may be critical for orienting the catalytic Cys189 residue and thereby facilitate the switching between protease and ligase activities. While molecular details differ, our findings are reminiscent of the mechanistic principles implemented by the catalytic tetrad in serine proteases, where the tetrad residue Ser214 (chymotrypsinogen numbering) is conserved over different serine protease clans and considered as an example of convergent evolution. In serine proteases, the tetrad residue interacts with the Asp102 of the Ser195-His57-Asp102 triad [24]. Thereby, it exerts activity-modulating effects [25].

Mutagenesis studies also revealed the relevance of residue Asn42 for legumain activity. Importantly, caspases, paracaspases and metacaspases harbor a carbonyl oxygen at the equivalent position. Therefore, it is not possible to directly access the function of this oxygen by introducing point mutations, because the carbonyl oxygen would always remain in place. Gingipain K however harbors an aspartate at the equivalent position. A corresponding mutation in legumain resulted in a pH-dependent reduction in protease activity and a significantly reduced ligase activity. A N42A-legumain mutant however proofed beneficial for ligase activity.

Taking these findings together, we suggest a refined catalytic mechanism of clan CD proteases that is based on a “catalytic dyad +”. The dyad consists of the strictly conserved cysteine and histidine residues, and is extended by the regulatory residues Asn42, Glu190 and Ser215. Furthermore, we previously identified Asp147 as another critical regulator of legumain activity [10]. The functional understanding of these regulatory residues allows us to tune the activity of a certain clan CD protease by introducing specific point mutations around the catalytic residues. This knowledge gives us the possibility to engineer proteases with higher enzymatic activity as their natural analogs.

Another important aspect we could show within this study is that legumain’s activity is not only defined by its active site, but it is also dependent on features of the substrate. Using human cystatin M/E as a model substrate, we found that not only the sequence, but also the geometry and dynamics of a substrate determine its turnover by legumain. While a rigid substrate favors ligation over hydrolysis, a flexible substrate favors hydrolysis rather than ligation (Figure 6). This conclusion can also be rationalized by the change in entropy, and thus free energy, of the substrate upon turnover. The rigid conformation of cystatin M/E is established by a conserved Lys75 residue. Following cleavage, Lys75 will favor the re-ligation of the free termini by keeping the N-terminus of the prime side cleavage product in close proximity to the C-terminus of the non-prime cleavage product. Additionally, Lys75 is also critical for the correct positioning of the legumain exosite loop (LEL), which is essential to establish a stable enzyme-inhibitor complex.

These findings open up new possibilities for the design of legumain inhibitors and ligase substrates. We suggest that conformationally locked peptidic inhibitors will prohibit cleavage by legumain. Similarly, non-covalently linking non-prime and prime side substrates will enforce the proximity of the free termini within the legumain active site, and thereby generate favorable ligase substrates. Importantly, this principle is also implemented in plant-derived cyclic peptides such as the sunflower trypsin inhibitor, which is cyclized by legumain.

## 4. Materials and Methods

### 4.1. Cloning

Full length prolegumain cDNA was cloned into the pLEXSY-sat2 expression vector using XbaI and KpnI restriction sites as described earlier [26]. The expression construct carried an N-terminal signal sequence for secretory expression and a C-terminal His_6_-tag. Expression constructs of human caspase-9 were prepared as described previously [10,27]. The construct carried the catalytic domain of human caspase-9 and a C-terminal His_6_-tag (∆CARD-caspase 9). Similarly, human cystatin M/E was cloned into the pET-22b(+) expression vector for periplasmic expression as described elsewhere [11].

Point mutants were generated using round-the-horn site directed mutagenesis, which is based on the inverse PCR method, as described elsewhere [28,29]. Correctness of the clones was verified via DNA-sequencing by Eurofins Genomics (Ebersberg, Germany).

### 4.2. Expression and Purification of Human Legumain

Expression, purification and autocatalytic activation of human prolegumain was done as described previously [26]. In brief, the full length prolegumain expression construct carrying a 6x-histidine tag at the C-terminus was transfected into the LEXSY P10 host (*Leishmania tarentolae* expression system, Jena Bioscience, Jena, Germany). Positive clones were selected using nourseothricin (Jena Bioscience). Prolegumain was expressed as secreted protein using cells grown in brain heart infusion (BHI) medium (Carl Roth, Karlsruhe, Germany) supplemented with 5 µg/mL Hemin (Applichem, Darmstadt, Germany), 50 units/mL Pen-Strep (Penicillin-Streptomycin, Carl Roth) and 0.1 mg/mL nourseothricin (NTC). Expression cultures were incubated shaking (140 rpm) at 26 °C until an OD600 of approx. 3 was reached. BHI medium containing recombinant prolegumain was harvested by centrifugation (17,500× *g*, 15 min, 4 °C) to remove cells and cell debris.

Subsequently, the supernatant was supplemented with 5 mM β-mercaptoethanol (Merck, Darmstadt, Germany) and incubated with Ni^2+^-NTA Superflow resin (Qiagen, Hilden, Germany) at 4 °C for 1 h. The Ni^2+^ beads were washed using a buffer composed of 20 mM Tris pH 7.0, 50 mM NaCl and 2 mM DTT and bound protein was eluted using wash buffer supplemented with 250 mM imidazole. Elutions were concentrated using Amicon Ultra centrifugal filter units (MWCO: 10 kDa; Cytiva, Freiburg im Breisgau, Germany) and buffer exchanged via PD-10 columns (Cytiva) to get the protein in the final buffer 20 mM Tris pH 7.0, 50 mM NaCl and 2 mM DTT. For subsequent assays, prolegumain was activated to the asparaginyl-specific endopeptidase (AEP) at pH 3.5 for 16 h at room temperature. For further purification, the activated AEP was loaded onto a Superdex 200 10/300 GL size exclusion chromatography column (GE Healthcare) which was pre-equilibrated in a buffer composed of 20 mM citric acid pH 4.0, and 50 mM NaCl.

### 4.3. Expression and Purification of Human Cystatin M/E

Human cystatin M/E wild-type and the hCE-K75A mutant were expressed to the periplasmic space of *E. coli* Bl21(DE3) cells as previously described [11]. Positive clones were selected using ampicillin. Expression cultures were grown in Luria-Bertani (LB) medium supplemented with 100 µg/mL of ampicillin at 37 °C with shaking at 230 rpm until an OD600 of ~0.8 was reached. Expression was induced upon addition of 1 mM Isopropyl β-D-1-thiogalactopyranoside (IPTG) at 25 °C. Following overnight incubation at 25 ° cells were harvested by centrifugation (10 min, 4000× *g*, 4 °C) and pellets were frozen at −20 °C. The periplasmic fraction was extracted by cold osmotic shock. Specifically, the frozen pellet was resuspended in cold extraction buffer containing 20% sucrose and 20 mM Tris pH 7.5 and stirred for 20 min at room temperature. Subsequently the solution was centrifuged for 20 min at 17,500× *g* and 4 °C. The resulting supernatant was subjected to Ni^2+^-affinity purification as described previously [11]. Elutions were concentrated using Amicon Ultra centrifugal filter units (MWCO: 3 kDa, Cytiva) and subjected to size exclusion chromatography utilizing a Superdex 75 10/300 GL column (Cytiva) pre-equilibrated in buffer composed of 20 mM citric acid, pH 5.5, and 100 mM NaCl.

### 4.4. Expression and Purification of Human ΔCARD Caspase-9

∆CARD-caspase 9 was expressed in *E.coli* BL21(DE3) cells as described previously [10,27]. Briefly, *E.coli* BL21(DE3) cells were transformed with the ∆CARD-caspase-9 expression plasmid. Positive clones were selected using ampicillin. Expression cultures were grown in LB medium supplemented with 100 µg/mL ampicillin at 37 °C with shaking at 230 rpm until an OD600 of approx. 0.7 was reached. Subsequently, expression cultures were transferred to 25 °C and expression was induced by addition of 0.4 mM IPTG. After 4 h, cultures were harvested by centrifugation (4000× rpm, 10 min, 4 °C), the pellet was resuspended in lysis buffer composed of 50 mM Tris pH 7.5 and 100 mM NaCl and lysed by sonication (3×, 45 s, 40% power, 50% cycle). Following centrifugation (17,500× *g*, 15 min, 4 °C), the supernatant was subjected to Ni^2+^-affinity purification as described previously [10]. Elutions were concentrated and subjected to size exclusion chromatography using a Superdex 75 10/300 GL column (Cytiva) pre-equilibrated in buffer composed of 20 mM Hepes pH 7.5, and 100 mM NaCl.

### 4.5. Enzymatic Activity Assays

The proteolytic activity of legumain was monitor using the fluorogenic Z-Ala-Ala-Asn-7-amino-4-methylcoumarin (AAN-AMC; Bachem, Bubendorf, Switzerland) substrate and the chromogenic Bz-Asn-4-nitroaniline (Bz-Asn-pNA; Bachem) substrate. Specifically, a substrate solution containing 50 mM citric acid pH 5.5, 100 mM NaCl, 0.05% Tween-20 and 50 µM AAN-AMC was prepared and the reaction started upon addition of 2 nM legumain. The increase in fluorescence signal was measured at 460 nm after excitation at 380 nm in an Infinite M200 Plate Reader (Tecan, Männedorf, Switzerland) at 37 °C. Alternatively, a substrate solution containing 50 mM citric acid pH 5.5 or 4.0, 100 mM NaCl, 0.05% Tween 20, 2 mM DTT and 200 µM Bz-Asn-pNA was used. End-point product absorption was measured at 405 nm and 37 °C. Legumain inhibition by cystatin M/E and the cystatin M/E-K75A mutant was assessed by addition of 4 nM of inhibitor before adding the enzyme.

The activity of caspase-9 was assayed in a buffer composed of 0.5 M Na_3_ citrate, 50 mM Hepes pH 7.5, 100 mM NaCl, and 0.05% Tween-20 supplemented with 100 µM Z-Val-Ala-Asp-AMC (VAD-AMC; Bachem) substrate. After addition of 1 µM enzyme, activity was measured at 37 °C as an increase in fluorescence at 460 nm upon excitation at 380 nm. K_M_ values of wild-type ΔCARD-caspase-9 and the ΔCARD-caspase-9-E288K mutant were determined by decreasing substrate concentration from 1 mM to 31 µM via a 1:2 dilution series. K_M_ values were calculated from initial velocities using GraphPad Prism version 9.3.1 for Windows (GraphPad Software, San Diego, CA, USA, www.graphpad.com, accessed on 17 October 2022).

### 4.6. Ki Determination

To determine the Ki of the hCE-K75A mutant towards legumain, the inhibitor was mixed with substrate solution (50 µM AAN-AMC in 20 mM citric acid pH 5.5, 50 mM NaCl and 0.05% Tween-20) at increasing concentrations ranging from 1.5 nM till 1.6 µM. The reaction was started upon addition of 2 nM legumain. The increase in fluorescence was measured for 10 min at 37 °C. A K_i_ was determined from the initial velocities using the Morrison equation implemented in GraphPad Prism version 9.3.1 for Windows (GraphPad Software, San Diego, CA, USA, www.graphpad.com).

### 4.7. Testing pH-Dependent Cleavage of Cystatin M/E by Legumain

Active legumain (13.8 µM) was incubated with cystatin M/E or the cystatin M/E-K75A mutant (28 µM) in a 1:2 molar ratio in a buffer containing 20 mM citric acid (pH 4.0–pH 6.0) or 20 mM Tris (pH 7.0) and 50 mM NaCl for 1 h at 37 °C. Subsequently, samples were analysed by SDS-PAGE using non-reducing sample loading buffer.

### 4.8. Testing Re-Ligation of Cystatin M/E and the Cystatin M/E-K75A Mutant by Legumain

Active legumain (13.8 µM) was incubated with the cystatin M/E-K75A mutant (28 µM) in a 1:2 molar ratio in a buffer containing 20 mM citric acid pH 4.0 and 50 mM NaCl for 1 h at 37 °C. Subsequently, aliquots of 10 µL were subjected with 100 mM of a Tris pH 7.0 stock solution or 2 mM S-methyl methanethiosulfonate (MMTS). After 1 h incubation at 37 °C, the reactions were stopped by the addition of 2 mM of the covalent Ac-Tyr-Val-Ala-Asp-chloromethylketone (YVAD-cmk; Bachem) inhibitor. Samples were analyzed by SDS-PAGE using non-reducing sample loading buffer.

### 4.9. Testing Cleavage and Re-Ligation of Cystatin M/E by Different Legumain Variants

Legumain and legumain mutants (10.4 µM) were incubated with wild-type cystatin M/E (3.1 µM) in a 3:1 molar ratio at pH 4.0 for 30 min at 37 °C. Subsequently, the reactions were split into (a) a control reaction which was supplemented with 0.5 mM YVAD-cmk, (ii) a reaction which was supplemented with 10 mM MMTS and (iii) a reaction where pH was shifted to neutral upon addition of 100 mM Tris pH 7.0. After 10 min of incubation at 37 °C, reactions (ii) and (iii) were stopped by the addition of 2 mM of the Ac-Tyr-Val-Ala-Asp-cmk inhibitor and samples were analyzed by SDS-PAGE. Bands corresponding to intact cystatin M/E and to the C-terminal cleavage product hCE(S40-M120) were quantified using ImageJ [30].

### 4.10. Thermal Shift Assay

A thermal shift assay was performed following previously published protocols [11,31]. Briefly, legumain was mixed with assay buffer containing 100 mM citric acid pH 6.0, 100 mM NaCl and 5x Sypro orange dye (Invitrogen) to a final concentration of 0.2 mg/ml. Thermal unfolding was measured by gradually increasing temperature from 20 °C to 90 °C in 1-degree steps. The fluorescence signal was detected using a 7500 Real Time PCR System (Applied Biosystems, Waltham, MA, USA). Fluorescence values were normalized to peak values and melting curves were evaluated following the protocol by F. Niesen [32].

### 4.11. Size Exclusion Chromatography Experiments

Complex formation of legumain with the hCE-K75A mutant was analyzed using co-migration assays. Specifically, legumain was incubated with the hCE-K75A mutant in a 1:4 molar ratio in a buffer composed of 50 mM citric acid pH 4.0 and 100 mM NaCl for 1 h at 37 °C. Then, the reaction was subjected to size exclusion chromatography using an ÄKTA FPLC system (Cytiva) equipped with a Superdex 75 10/300 GL or Superdex S200 10/300 GL column pre-equilibrated in buffer containing 20 mM citric acid pH 4.0 and 50 mM NaCl. Peak fractions were analyzed by SDS-PAGE.

### 4.12. Preparation of Sequence Alignments

The structure-based alignment shown in Figure 1 was created using Topmatch [33]. The alignment of human caspase sequences shown in Figure 3 was created using ClustalW [34] using the following protein sequences (UniProt IDs): Caspase-1: P29466, Caspase-2: P42575, Caspase-3: P42574, Caspase-4: P49662, Caspase-5: P51878, Caspase-6: P55212, Caspase-7: P55210, Caspase-8: Q14790, Caspase-9: P55211, Caspase-10: Q92851, Caspase-14: P31944.

The sequence alignment shown in Figure 4 was prepared using ClustalW using the following sequences (Uniprot IDs): human legumain (Q99538), mouse legumain (O89017), jack bean legumain (P49046), Vacuolar-processing enzyme beta-isozyme 1 from *Oryza sativa* (OsVPE1; Q84LM2), *Helianthus annuus* asparaginyl endopeptidase 1 (HaAEP1; A0A0G2RI59), peptide asparaginyl ligase 2 from *Viola philippica* (VpPAL2; A0A4Y5MSQ3) legumain isoform beta from *Arabidopsis thaliana* (AtAEPβ; Q39119), Asparaginyl endopeptidase 3a and 3b from *Petunia hybrid* (PxAEP3A; and PxAEP3b; A0A2S1GGU3) and phosphatidylinositol-glycan biosynthesis class K protein (PIGK; Q92643).

The sequence alignment shown in Figure 5 was prepared using ClustalW using the following sequences (Uniprot IDs): human cystatin M/E (Q15828), human cystatin C (P01034), human cystatin F (O76096), human cystatin D (19882256), human cystatin S (001890.1), human cystatin SA (001313.1), human cystatin SN (19882251), chimpanzee cystatin M/E (A0A2R8Z8E8), *M. mulatta* cystatin M/E (H9F6U7), rat cystatin E/M (A0A8I5ZXE3), rat cystatin C (P14841), rat cystatin F (D3ZCV2), mouse cystatin E/M (Q15828), mouse cystatin C (P21460) and mouse cystatin F (O89098).

### 4.13. Preparation of Figures Illustrating Protein Structures

Figures illustrating protein structures were prepared using the PyMOL Molecular Graphics System, Version 2.0 Schrödinger, LLC [35].

## Data Availability

Not applicable.

## Supplementary Materials

The following supporting information can be downloaded at: https://www.mdpi.com/article/10.3390/ijms232012548/s1.
