# Supplementary material for: Legumain Activity Is Controlled by Extended Active Site Residues and Substrate Conformation
Source: Int J Mol Sci. 2022 Oct 19;23(20):12548. doi: 10.3390/ijms232012548 (PMC9604545; doi:10.3390/ijms232012548)
Supplement: Supplementary file 1 [file ijms-23-12548-s001.zip › ijms-1935404-supplementary.pdf]

Legumain Activity Is Controlled by Extended Active Site Residues and Substrate Conformation

Tasneem Elamin [#], Hans Brandstetter [#], Elfriede Dall [#,†]

Affiliation

#Department of Biosciences and Medical Biology, University of Salzburg, 5020 Salzburg, Austria

Corresponding Author

[†] Elfriede Dall (elfriede.dall@plus.ac.at)

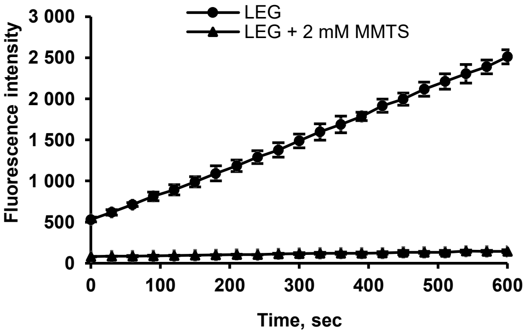

Supplementary Figure S1. The catalytic Cys189 on legumain is efficiently blocked by MMTS. The turnover of the AAN-AMC substrate by legumain was measured, with or without pre-incubation with 2 mM MMTS. MMTS-treated legumain did not show substrate turnover, which confirmed that the catalytic Cys189 residue was efficiently modified by MMTS.

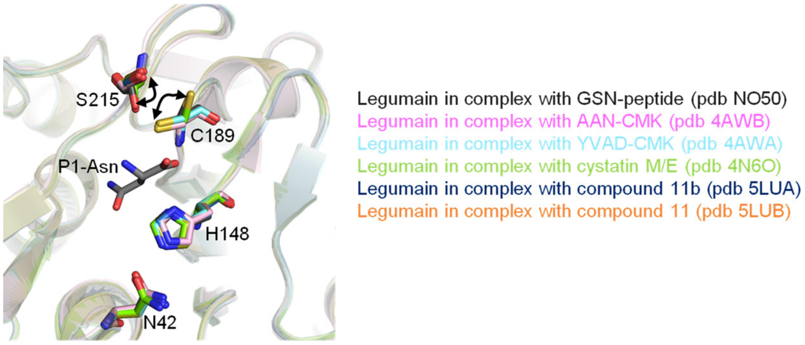

Supplementary Figure S2. Cys189 and Ser215 adapt distinct orientations in different structures of legumain. Superposition of structures of legumain in complex with different ligands. Grey: legumain in complex with the Gly-Ser-Asn-peptide (pdb NO50), pink: legumain in complex with AAN-CMK (pdb 4AWB), light blue: legumain in complex with YVAD-CMK at pH 5.0 (pdb 4AWA), green: legumain in complex with cystatin M/E (pdb 4N6O), dark blue: legumain in complex with compound 11b (pdb 5LUA), orange: legumain in complex with compound 11 (pdb 5LUB).

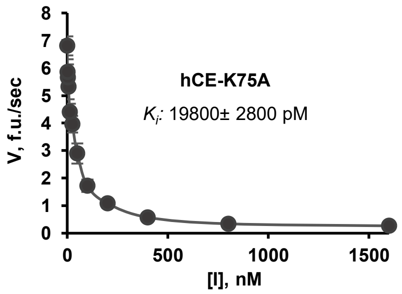

Supplementary Figure S3. The K_i of the cystatin M/E-K75A mutants towards legumain was determined using Morrison's equation. Activity was measured at pH 5.5 using the Z-Ala-Ala-Asn-AMC legumain specific substrate.
